# A proposed technique for robotic port placement and arterial control of the femoral region for a peripheral arterial bypass with the Da Vinci SP system

**DOI:** 10.1007/s11701-026-03664-3

**Published:** 2026-07-27

**Authors:** Philip Stather, A. J. Williamson, G. Stante, L. Ryckembusch, P. Bhandari, A. Q. Howard

**Affiliations:** 1 Norwich Vascular Unit, Norfolk and Norwich University Hospital, Norwich, UK; 2 Norwich Medical School, University of East Anglia, Norwich, UK; 3 Section of Vascular Surgery, The University of Chicago Medicine, Chicago, IL USA; 4 Intuitive Surgical Inc, Sunnyvale, CA USA; 5 Department of Vascular Surgery, Colchester General Hospital, Turner Road, Colchester, UK

**Keywords:** Vascular, Femoral, Robotic, Clamping, Arterial

## Abstract

Robotic surgery has expanded minimally invasive options across several surgical specialties, yet adoption in peripheral vascular surgery remains limited. Groin incisions carry a high risk of wound infection with potentially devastating consequences. The da Vinci Single Port (SP) surgical system enables development of a subcutaneous space, relocating the incision away from the femoral region and potentially reducing wound complications. Earlier cadaveric studies identified arterial control and port placement as challenges for robotic femoral access. Additional iterative cadaveric sessions were undertaken to optimise these parameters. Each session assessed port positioning, exposure of femoral vessels, and feasibility of vascular reconstruction and arterial control. Initial medial mid-thigh port placement resulted in limited visualisation, extended dissection, or frequent robotic arm collision with the lower limb. Lateral repositioning to the ventral thigh improved visualization of the femoral triangle and eliminated external robotic arm interference. Centring the port 5 cm caudal to the femoral bifurcation on the ventral aspect of the thigh provided a reproducible anatomical landmark. Utilizing this configuration, robotic dissection of the arteries was consistently achieved, and vascular reconstruction was feasible. Arterial control strategies were evaluated, with percutaneous vascular clamping under robotic visualisation, slings with a vascular tourniquet, or endovascular control all being feasible techniques. Lateral ventral-thigh port placement avoids robotic arm collision with the patient and facilitates vascular reconstruction. Percutaneous clamping provides a practical strategy for arterial control with slings and endovascular approaches viable alternatives. These technical parameters provide a foundation for future clinical evaluation of robotic peripheral vascular surgery.

**Supplementary Information:**

The online version contains supplementary material available at 10.1007/s11701-026-03664-3.

## Introduction

Robotic surgery has transformed many surgical procedures by combining the ergonomic and visual advantages of minimally invasive surgery with enhanced dexterity, and high-definition three-dimensional visualisation. These features allow precise dissection and suturing within confined anatomical spaces and have contributed to the widespread adoption of robotic surgery in urology, gynaecology and general surgery [1].

The potential role of robotics in vascular surgery is in the ability to combine the durability of open reconstruction, with the reduced morbidity associated with minimally invasive procedures. Within the multiport field, early reports have highlighted feasibility in aortic aneurysm repair, aorto-iliac reconstruction, visceral artery interventions, and venous surgery. However, uptake of these remains limited to a small number of pioneering centres, with challenges of training in an unfamiliar robotic system, adequate vessel exposure and safe reconstruction limiting widespread adoption [2–5].

Conventional multi-port robotic systems require wide port triangulation, which is difficult to achieve in the confined femoral region due to the proximity of the lower limb and the limited working distance between the skin and target vessels. The da Vinci Single Port (SP) surgical system enables dissection and anastomosis within a confined space by delivering a flexible three-dimensional endoscope and up to three wristed instruments through a single incision, while reducing external arm interference as compared to multi-port systems. It also enables incision placement away from the anastomotic site, potentially lowering the risk of wound and subsequent anastomotic infections. This architecture provides the potential for improved access to narrow anatomical corridors and may enable robotic approaches to vascular regions.

Early cadaveric work has begun to explore the feasibility of single-port robotic vascular exposure [6]. The findings suggest that SP may overcome some of the spatial limitations that have hindered robotic peripheral vascular surgery. Despite these early demonstrations of feasibility, several critical technical questions remain unresolved. In particular, optimal port site placement, maintenance of an adequate operative view within the confined femoral region, avoidance of robotic arm collision with the lower limb, and the ability to safely obtain proximal and distal vascular control are fundamental requirements for safe robotic vascular surgery. The geometry of the femoral triangle and the presence of the lower extremity introduce unique constraints that may influence instrument angles, visualisation, and surgeon ergonomics when using a single-port robotic platform.

The aim of this study was therefore to optimise port placement and arterial control for robotic femoral vascular exposure using the da Vinci SP system in a cadaveric model, with a specific focus on port site placement, instrument articulation within the confined femoral region, and the ability to achieve reliable inflow and outflow control of the femoral vessels, for a peripheral arterial bypass. By defining these technical parameters, this study seeks to provide foundational data to guide the development of robotic approaches to peripheral vascular surgery.

## Methods

### Study design

The study was designed as a cadaveric feasibility study to evaluate the technical parameters required for robotic femoral vascular exposure using the da Vinci SP surgical system (Intuitive Surgical, Sunnyvale, CA, USA). The primary focus of the study was to assess optimal port site placement, instrument articulation within the confined femoral region, and the ability to achieve reliable proximal and distal vascular control of the femoral vessels.

All procedures were performed in a cadaveric laboratory environment using fresh frozen cadaveric specimens. The study aimed to simulate the anatomical and spatial constraints encountered during femoral arterial exposure in clinical practice.

### Ethics

This study utilised cadaveric specimens obtained through an approved anatomical donation programme. As no living human participants were involved, institutional review board approval was not required.

### Cadaver positioning

Cadaveric specimens were positioned supine on the operating table, with the ipsilateral edge of the torso aligned to the lateral boundary of the operating table. The lower limb was placed in a neutral extended position to replicate the positioning typically used during open femoral artery exposure.

The ipsilateral leg was positioned to allow realistic assessment of the spatial relationship between the robotic platform and the lower extremity. Particular attention was paid to potential external robotic arm collision with the lower limb during docking and instrument manipulation. The SP patient cart was situated at the contralateral side of the bed centred around the patient umbilicus.

### Robotic platform

All procedures were performed using the da Vinci SP surgical system. This platform utilises a single 4 cm incision and access port through which a flexible three-dimensional endoscope and three multi-jointed wristed instruments are deployed. The system allows internal articulation of the instruments distal to the incision, enabling triangulation within confined operative spaces while minimising external robotic arm movement.

For this study, standard SP robotic instruments were used, including Monopolar Curved Scissors (MCS), Fenestrated Bipolar Forceps (FBF), and Cadiere Forceps to allow tissue dissection. Additionally, a Needle Driver was used for suturing tasks. Practical and theoretical demonstration of arterial control was undertaken using slings, clips, clamps and occlusion balloons.

### Port site placement

Port site placement was evaluated with the aim of identifying an optimal position out of the groin crease that allowed safe access to the femoral vessels while maintaining adequate visualisation and avoiding interference from the lower limb.

A single SP small access port was placed in the proximal thigh, positioned to provide a direct trajectory towards the femoral triangle. The distance from the port to the inguinal ligament was measured to ensure sufficient working length for instrument deployment and articulation.

**Fig. 1:**
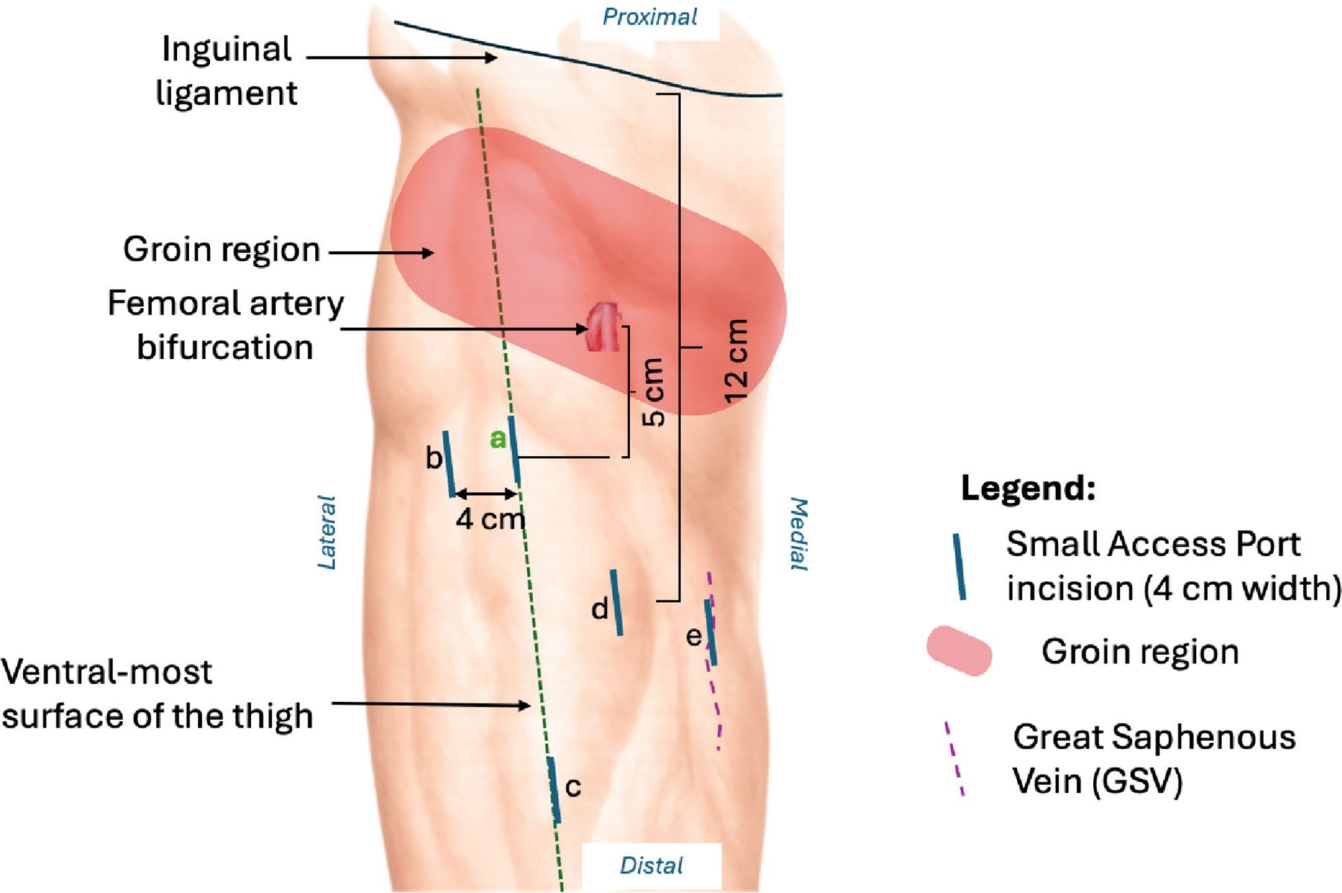
Schematic for all incision locations for the da Vinci SP small Access Port (AP) explored in this study: (**a**) 5 cm caudal to the femoral bifurcation on the ventral-most surface of the thigh; (**b**) 5 cm caudal from femoral bifurcation and 4 cm lateral to the ventral-most surface of the thigh; (**c**) mid-thigh on the ventral-most surface of the thigh; (**d**) 12 cm inferior to the inguinal ligament, directly over the superficial femoral artery; (**e**) mid-thigh directly over the greater saphenous vein (GSV)

Different port positions were evaluated to assess instrument reach to the femoral vessels, quality of visualisation of the femoral triangle, ability to undertake an anastomosis, and potential collision between the SP Access Port or instruments and the lower limb (Fig. 1).

### Robotic femoral dissection

Following docking, robotic instruments were deployed and dissection of the femoral region was performed under three-dimensional robotic visualisation. Robotic instruments were used to mobilise the vessels and develop circumferential control while maintaining clear visualisation of surrounding structures.

### Technical evaluation

Technical feasibility was evaluated according to the following criteria:

- adequacy of visualisation of the femoral vessels to enable safe dissection up to and beyond the inferior epigastric and circumflex branches, and of the superficial femoral and Profunda femoris arteries.
- ability to perform precise robotic dissection along both medial and lateral sides of the arteries minimising risk of damaging branches.
- avoidance of robotic arm collision with the lower limb.
- ability to achieve reliable proximal and distal vascular control through any technique as described within the arterial control section.
- feasibility of performing a vascular anastomosis with a graft or patch, and technical difficulty in achieving the same.

Observations regarding optimal port placement and instrument positioning were recorded during each procedure.

## Results

### Port site positioning

Prior to determining port site position, the anatomy of the leg was outlined, with the inguinal ligament identified, and leg orientated into its natural position. The route of the femoral artery was identified using ultrasound, and the femoral bifurcation was marked. The most ventral aspect of the thigh was also marked, and the course of the great saphenous vein was marked using ultrasound (Fig. 1).

Initial labs utilised a range of port sites (Table 1). The scale and grading were decided upon consensus with 3 surgeons across ~ 27 cadaver lab attempts (Supplementary Table 1) in total. Port sites B-E suffered difficulties in particular with collision between the robotic instrument cluster and the patient’s leg. Despite good ability to dissect with most approaches, a more lateral approach as with site B (Supplementary Fig. 1) hindered visualisation and vessel control. A medial or distal approach as with sites C-E (Supplementary Figs. 2–4) incurred significant collisions, and hampered visualisation for the anastomosis.

**Table 1:** Showing each port site placement and its score with key technical evaluation. Each component was scored out of 5

Port Site*	Visualisation	Dissection	Avoidance of Arm Collision	Vessel Control	Anastomosis
A (Fig. 2)	5	5	5	5	5
B (Supplementary Fig. 1)	3	4	5	3	4
C (Supplementary Fig. 2)	5	4	3	4	3
D (Supplementary Fig. 3)	3	4	2	4	2
E (Supplementary Fig. 4)	3	4	1	5	3

*See Fig. 1 for description and location of each port site

**Fig. 2:**
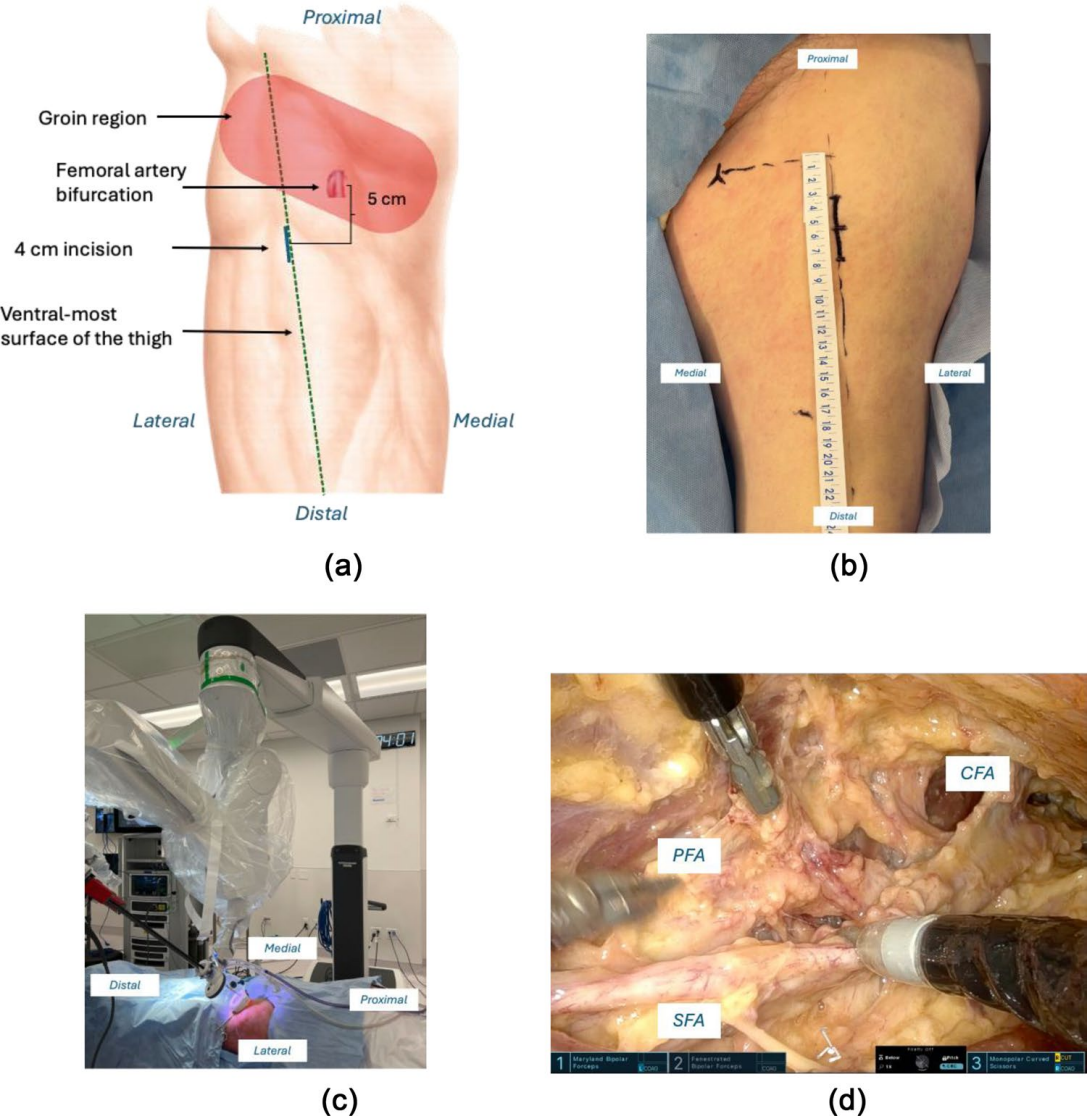
Small AP incision 5 cm from femoral bifurcation on the ventral-most surface of the thigh. Identified as the most optimal incision location. **a** SP Small AP incision 5 cm from femoral bifurcation on the ventral-most surface of the thigh (Right leg shown). Identified as the ideal incision location. **b** SP Small AP incision 5 cm from femoral bifurcation on the ventral-most surface of the thigh (left leg). Identified as the ideal incision location. **c** External view with instrument tips at the femoral bifurcation, resulting in no patient-robotic arm collisions (left leg operation). **d** Endoscopic view with instrument tips at the femoral bifurcation (left leg)

Subsequently, the access port was repositioned to remove the risk of collision with the leg, whilst providing optimal visualisation, dissection and control. This site is defined as 5 cm caudal to the femoral bifurcation and positioned laterally onto the most ventral (closest to the ceiling in a supine position) aspect of the thigh (Fig. 2a, b). This spot equates to the centrepoint of the incision.

This approach was subsequently validated in 15 further cadaveric lab sessions (Supplementary Table ). No external robotic arm collision was observed (Fig. 2).

### Femoral exposure and anastomosis

In all lab simulations dissection of the common femoral artery up to and beyond the inguinal ligament was achieved successfully. In addition, dissection of the superficial femoral artery and profunda artery was also achieved.

Due to limitations of the initial port sites as above, we will describe the dissection and approach to the femoral vasculature from the final port location (A). The location of the incision is directly over the sartorius muscle. The initial 4 cm skin incision is continued deep to identify the fascia lata. The muscle fibres of the sartorius will be visible through the fascia lata. At this layer, a finger sweep is performed to blunt dissect a 2–3 cm border around the incision site to enable placement of the access port. Following access port insertion and docking of the da Vinci SP, dissection continues perpendicular to the lines of the sartorius muscle fibres medially. This will enable identification of the superficial femoral artery. Following identification of the superficial femoral artery the dissection continues proximally to identify the femoral bifurcation and continues to the level of the inguinal ligament and deep circumflex and inferior epigastric vessels. In one lab session this dissection was continued proximally along the external iliac artery to identify and control the internal and common iliac arteries.

Through all cadaveric sessions, vascular anastomosis of either a bovine patch or prosthetic graft was achieved using a shortened 5 − 0 polypropylene (Prolene) or expanded polytetrafluoroethylene (Gore-Tex) sutures. Unlike initial incision sites (C, D, and E) which demanded that the anastomosis start at the most proximal aspect of the arteriotomy, the final port site incision enabled anastomosis at either the heel, or 3 O’clock position due to a more natural lateral angle of the camera and instruments.

### Arterial control

**Fig. 3:**
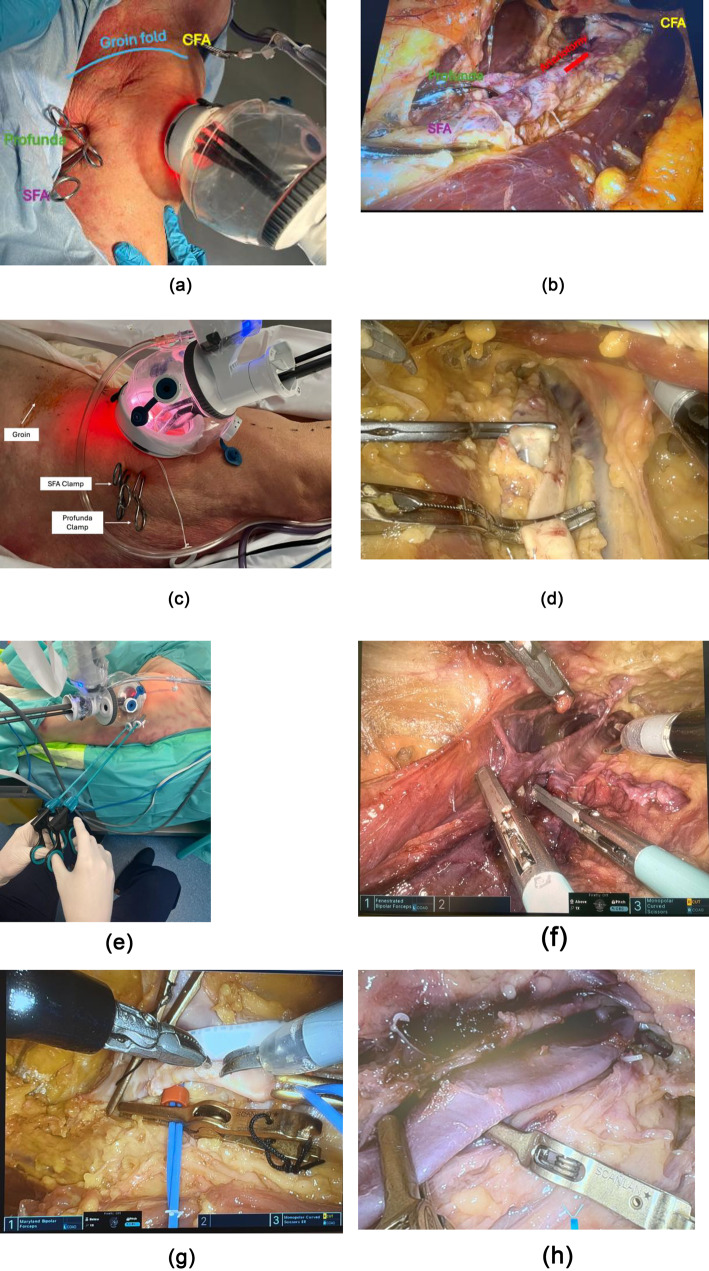
Various techniques for arterial vascular control: **a, b**: Percutaneous clamps introduced from the medial side of the thigh; **c, d**: Percutaneous clamps introduced from the lateral side of the thigh; **e, f**: Laparoscopic clamps introduced from the lateral side of the thigh through laparoscopic ports; **g**: Rumel tourniquet utilized for clamping using a vessel loop around end pieces of a rubber tube; **h**: low pressure drop-in bulldog clamps.** a**: External view of clamping locations. Two percutaneous stab incisions (adjacent to each other) were made with a #11 blade through the medial aspect of the thigh at the level at or below the SP incision to introduce profunda clamps or paediatric DeBakey clamps for clamping the profunda and SFA. This medial thigh location for the distal clamping resulted in no interference of the clamps with the robotic instruments (neither internal nor external). A stab incision with a no. 11 blade on the groin fold in the same line as the SP incision was used to introduce the proximal clamp for control above the CFA.** b**: Corresponding endoscopic view of vascular clamp control with the distal clamps introduced through the medial aspect of the thigh. No interference with the clamps was observed for the SP instruments. **c**: Alternatively, a similar location on the level at or below the SP access port, but on the lateral aspect of the thigh provides similar results for distal clamping of the profunda and SFA. **d**: Corresponding endoscopic view of the profunda and SFA clamped via percutaneous incisions lateral to the SP access port. No interference with the clamps was observed for the SP instruments. **e**: Laparoscopic port placement for clamp localisation. **f**: Laproscopic clamp placement. **g**: Clamping of femoral vessels using vessel loops around end pieces of a rubber tube (Rumel tourniquet). **h**: Low pressure drop-in bulldog clamps (ScanLan Inc.) for vascular control

Without optimal arterial control, vascular intervention robotically will not be possible. Several options for this have been postulated, including balloon control, slings, and clamps. This section will compare and contrast these approaches (Fig. 3; Table 2).

**Table 2:** Arterial control methods evaluated and their advantages, limitations, and anticipated clinical applicability

Arterial control method	Advantages	Limitations	Anticipated clinical applicability
Endovascular balloon occlusion	• Well described and frequently performed technique • Completely endoluminal; eliminates external clamps/slings; does not clutter surgical field • No circumferential dissection required	• Difficult to simultaneously block retrograde flow from multiple branches without multiple balloon catheters. • C-arm or hybrid OR required	Proximal control of external iliac artery/SFA.
Vessel Loop	• Low profile • Gentle on tissues • Excellent for retraction	• Manual tensioning required • Incomplete occlusion can occur in heavily calcified arteries • Circumferential dissection required	Baseline clamping mechanism for minimally calcified arteries and branches such as the circumflex artery
Rumel tourniquet	• Continuous vessel compression without manual hold • Dynamic adjustment to check patency or flush the artery	• Technically more challenging to perform • Circumferential dissection required • Bulky exterior components can clutter surgical field	Highly effective for deep, narrow exposures (such as isolating a deep PFA branch)
*Drop-in* Bulldog vascular clamps	• Autonomous vessel control i.e. *dropped-in* such that robotic arms are freed up • Atraumatic force minimizing risk of fracturing calcified arterial walls	• Limited force may fail to fully occlude heavily calcified arteries • Bulky in tight spaces	Optimal for reconstructions requiring maximum instrument autonomy
Percutaneous vascular clamping	• Reliable pressure • Minimal workspace footprint • Reproduces tactile reliability of standard open vascular clamps	• Fixed angles of approach require precise line-of-sight positioning • Risk of inadvertent torque • Risk of inadvertent injury to adjacent structures while introduction of clamps • Risk of insufflation loss	Optimal for reliable occlusion of severely calcified arteries without cluttering primary surgical field

Endovascular balloon occlusion of the external iliac artery can be obtained either from the contralateral side or brachial approach. Whilst suitable for proximal control, distal control is more complex. This would need to be obtained post arteriotomy and introduces an additional catheter within the operative field which may interfere with robotic instrument manipulation.

Vessel slings can be applied using a double sling technique, however applying sufficient force to the sling with the robotic instruments to occlude a perfused artery has not been tested. In typical open surgery vascular control is often achieved through clipping the sling to an external site. This can be achieved through percutaneous delivery of the slings; however this must be undertaken ensuring sufficient lateral or medial deflection to avoid creating obstacles within the dissection field. Alternatively, both ends of the sling can be passed through a short vascular tourniquet (Rumel) and a clip applied robotically (Fig. 3g).

Although bulldog vascular clamps (Fig. 3h) can be applied robotically and have been assessed in robotic nephrectomy [7], their ability to adequately control blood flow in calcified vessels containing plaque has not been established. In addition, orientation of the bulldog must be considered to ensure they don’t interfere with the robotic arms.

Percutaneous vascular clamping (Fig. 3a-d) under direct robotic visualisation provided the most reliable and reproducible method of arterial control during cadaveric testing. Percutaneous clamping can be applied under direct vision. Within the cadaveric labs, initially a needle was inserted to verify position and trajectory on the medial aspect of the groin, then a number 11 blade was used to create a skin incision (Fig. 3a, b). A paediatric DeBakey clamp or a Baby Vascular Clamp (ScanLan Inc.) was then positioned above the inferior epigastric and lateral circumflex branches under direct visualisation. Alternatively, the percutaneous clamps can be introduced from the lateral aspect of the thigh which also allows minimal collisions of the clamps with the robotic instruments and sufficient space and visualization of the anastomosis site (Fig. 3c, d). An alternative method was to use 5 mm laparoscopic ports and laparoscopic clamps (Fig. 3e, f). Control of the superficial femoral artery was similarly obtained using a small incision on the medial aspect of the thigh. Control of the profunda femoris can be obtained from either a lateral approach or medial approach (Fig. 3a-d). Further cadaveric labs undertaken using paediatric DeBakey clamps and vascular clamps confirmed reproducibility of this technique.

A combined approach to arterial control can be used with any of the above options used in combination, such as endovascular balloon control of the inflow, and clamping or slings for distal control (Table 2).

Lastly, in an emergency scenario, undocking protocols for the da Vinci SP platform are highly reproducible and supported by well-established safety benchmarks, taking under 90 s. Should control not be possible through percutaneous or other routes, the surgeon can simply make a new incision as standard directly over the CFA to obtain control.

## Discussion

This cadaveric study evaluated the technical feasibility of robotic femoral vascular exposure using the da Vinci SP surgical system and identified several key factors required for successful, uninhibited robotic access to the femoral vessels. In particular, the study defined reproducible parameters for port site placement, demonstrated the ability to undertake femoral artery dissection and reconstruction using the SP system, and evaluated several potential strategies for achieving arterial control.

The most important technical finding was the critical role of port site positioning. Initial medial port placement resulted in a distal approach to the femoral vessels with limited visualisation of the arterial lumen and frequent collision between the robotic arm and the lower limb. Through iterative cadaveric testing, repositioning the port laterally to the most ventral aspect of the thigh and 5 cm caudal to the femoral bifurcation significantly improved the viewing angle of key anatomical structures, facilitated vascular reconstruction, and eliminated robotic arm collision.

Using this final port position, robotic exposure of the femoral vasculature was consistently achieved. Dissection of the common femoral artery, superficial femoral artery and profunda femoris artery was possible within the confined femoral triangle, and vascular reconstruction using patch angioplasty or bypass grafting could be performed within the cadaveric model. Dissection up to the level of the common iliac artery was also feasible. These findings suggest that the SP system provides sufficient visualisation and instrument articulation to allow robotic dissection of the femoral vessels.

Achieving reliable arterial control remains a key challenge for robotic vascular surgery. Several potential approaches were explored during the cadaveric simulations. Endovascular balloon occlusion of the external iliac artery may provide proximal control but requires combination with a different technique for distal control and introduces X-rays and additional machinery into the procedure. Vessel slings can be applied robotically, however their ability to adequately control blood flow in vessels containing plaque and calcium has not been established. In this study, percutaneous vascular clamping under direct robotic visualisation provided the most reliable and reproducible method of arterial control. This approach allowed secure occlusion while maintaining unobstructed robotic instrument movement during vascular reconstruction and ensured the ability to backbleed and flush easily.

Our approach leverages the da Vinci SP’s ability to work through smaller incisions while maintaining a sufficient working space for complex dissection. The da Vinci SP system allows for precise and targeted dissection, which minimizes unnecessary tissue disruption. The incision size for the da Vinci SP robotic approach (4 cm) is smaller than standard open femoral exposure (10–12 cm) [8, 9]. Further, the robotic approach often requires less manual retraction and manipulation, potentially reducing trauma to surrounding tissues. Furthermore, strategies such as the use of tissue approximation techniques and, placement of drains or suction dressings) could be employed to mitigate seroma risks. The primary focus for our work was to remove the groin incision; the risk of seroma formation needs to be evaluated in the future.

Beyond the immediate technical implications of this work, establishing reproducible and safe robotic operative techniques represents an important foundation for the future evolution of robotic vascular surgery [10]. Standardised procedural workflows and validated operative approaches will be essential as the field progresses, particularly in view of the progress in the robotic field of remote surgery [11].

Several limitations of this study should be acknowledged. The cadaveric model does not replicate the presence of active bleeding, tissue perfusion or arterial pulsatility encountered during live surgery. In addition, the number of cadaveric sessions was limited and may not fully reflect the learning curve associated with robotic femoral exposure, especially with the level of disease encountered during a femoral endarterectomy compared to a cadaver. Quantitative procedural data were also limited at this early stage of technique development.

Despite these limitations, this study defines several important technical parameters for robotic femoral vascular surgery using the SP platform. The identification of optimal port geometry and a reproducible strategy for arterial control represents an important step toward clinical translation. The da Vinci SP surgical system is not currently indicated for vascular surgery globally and would require regulatory body and ethics committee approval to be studied clinically.

## Supplementary Information

Below is the link to the electronic supplementary material.

Supplementary Material 1

Supplementary Material 2

## Data Availability

No datasets were generated or analysed during the current study.
