# Supplementary material for: A proposed technique for robotic port placement and arterial control of the femoral region for a peripheral arterial bypass with the Da Vinci SP system
Source: J Robot Surg. 2026 Jul 27;20(1):756. doi: 10.1007/s11701-026-03664-3 (PMC13402264; doi:10.1007/s11701-026-03664-3)
Supplement: Supplementary file 2 — Supplementary Material 2 [file 11701_2026_3664_MOESM2_ESM.docx]

**A Proposed Technique for Robotic Port Placement and Arterial Control of the Femoral Region for a Peripheral Arterial Bypass with the Da Vinci SP System.**

Stather PW^1,2^, Williamson AJ^3^, Stante G^4^, Ryckembusch L^4^, Bhandari P^4^, Howard AQ^5^.

1 – Norwich Vascular Unit, Norfolk and Norwich University Hospital, Norwich, UK

2 – Norwich Medical School, University of East Anglia, Norwich, UK

3 – Section of Vascular Surgery, The University of Chicago Medicine, Chicago, IL USA

4 – Intuitive Surgical Inc., Sunnyvale, CA USA

5 – Department of Vascular Surgery, Colchester General Hospital, Turner Road, Colchester, UK

Corresponding Author: Philip Stather, ORCID-ID 0000-0002-3585-6728, email address: [p.stather@uea.ac.uk](mailto:p.stather@uea.ac.uk)

**SUPPLEMENTARY FIGURES:**

|   SP Small AP incision 5 cm from femoral bifurcation and 4 cm lateral to the ventral-most surface of the thigh |   SP Small AP incision 5 cm from femoral bifurcation and 4 cm lateral to the ventral-most surface of the thigh |
| --- | --- |
|   External view with instrument tips at the femoral bifurcation |   Endoscopic view with instrument tips at the femoral bifurcation |

**Supplementary Figure 1**: SP Small AP incision 5 cm from femoral bifurcation and 4 cm lateral to the ventral-most surface of the thigh

|   SP Small AP incision mid-thigh i.e. 14 cm from inguinal ligament |   SP Small AP incision mid-thigh i.e. 14 cm from inguinal ligament |
| --- | --- |
|   External view with instrument tips at the femoral bifurcation |   Endoscopic view with instrument tips at the femoral bifurcation |

**Supplementary Figure 2**: SP Small AP incision mid-thigh i.e. 14 cm from inguinal ligament

|   SP Small AP incision 12 cm from inguinal ligament and over the superficial femoral artery |   SP Small AP incision 12 cm from inguinal ligament and 3 cm medial to the ventral-most surface of the thigh |
| --- | --- |
|   External view with instrument tips at the femoral bifurcation |   Endoscopic view with instrument tips at the femoral bifurcation |

**Supplementary Figure 3**: SP Small AP incision 12 cm from inguinal ligament and 3 cm medial to the ventral-most surface of the thigh

|   SP Small AP incision mid-thigh on the greater saphenous vein (GSV) |   SP Small AP incision mid-thigh on the greater saphenous vein (GSV) |
| --- | --- |
|   External view of the robot and patient with the instrument tips of the robotic instruments at the femoral bifurcation |   Corresponding endoscopic view with the instrument tips at the femoral bifurcation |

**Supplementary Figure 4**: SP Small AP incision 6 cm from inguinal ligament on the greater saphenous vein (GSV)

**Supplementary Table 1**

| **No.** | **Incision location** | **Patient Side (Left/Right)** | **Port placement image** |
| --- | --- | --- | --- |
| 1. | 5cm from bifurcation | Left |  |
| 2. | 6 cm lateral and 3 cm superior to bifurcation | Left |  |
| 3. | 5cm from bifurcation | Left |  |
| 4. | 5cm from bifurcation | Left |  |
| 5. | 5cm from bifurcation | Left |  |
| 6. | 4cm from bifurcation | Right |  |
| 7. | 4cm from bifurcation | Right |  |
| 8. | 3cm from bifurcation | Left |  |
| 9. | 4cm from bifurcation | Left |  |
| 10. | 6cm from bifurcation | Left |  |
| 11. | 4cm from bifurcation | Right |  |
| 12. | 6cm from bifurcation | Left |  |
| 13. | 14 cm from inguinal ligament | Right |  |
| 14. | 12cm from inguinal ligament | Right |  |
| 15. | 15cm medial from inguinal ligament | Right |  |
| 16. | medial thigh | Right |  |
| 17. | 15cmfrom inguinal ligament | Right |  |
| 18. | 15cmfrom inguinal ligament | Left |  |
| 19. | 12cm from inguinal ligament | Right |  |
| 20. | 15cm from inguinal ligament | Right |  |
| 21. | 10cm from inguinal ligament | Left |  |
| 22. | 15cm from inguinal ligament | Left |  |
| 23. | 20cm from inguinal ligament, on GSV | Left |  |
| 24. | 15cm from inguinal ligament | Right |  |
| 25. | 20cm from inguinal ligament | Left |  |
| 26. | 25cm from inguinal ligament | Left |  |
| 27. | 20cm from inguinal ligament | Right |  |
